# Current Perspectives on the Use of Alternative Species in Human Health and Ecological Hazard Assessments

**DOI:** 10.1289/ehp.1306638

**Published:** 2013-06-14

**Authors:** Edward J. Perkins, Gerald T. Ankley, Kevin M. Crofton, Natàlia Garcia-Reyero, Carlie A. LaLone, Mark S. Johnson, Joseph E. Tietge, Daniel L. Villeneuve

**Affiliations:** 1 U.S. Army Engineer Research and Development Center, Vicksburg, Mississippi, USA; 2 National Health and Environmental Effects Research Laboratory, Mid-Continent Ecology Division, Office of Research and Development, U.S. Environmental Protection Agency, Duluth, Minnesota, USA; 3 National Center for Computational Toxicology, Office of Research and Development, U.S. Environmental Protection Agency, Research Triangle Park, North Carolina, USA; 4 Institute for Genomics, Biocomputing and Biotechnology, Mississippi State University, Starkville, Mississippi, USA; 5 U.S. Army Institute of Public Health, Health Effects Research Program, Aberdeen Proving Ground, Maryland, USA

## Abstract

Background: Traditional animal toxicity tests can be time and resource intensive, thereby limiting the number of chemicals that can be comprehensively tested for potential hazards to humans and/or to the environment.

Objective: We compared several types of data to demonstrate how alternative models can be used to inform both human and ecological risk assessment.

Methods: We reviewed and compared data derived from high throughput *in vitro* assays to fish reproductive tests for seven chemicals. We investigated whether human-focused assays can be predictive of chemical hazards in the environment. We examined how conserved pathways enable the use of nonmammalian models, such as fathead minnow, zebrafish, and *Xenopus laevis*, to understand modes of action and to screen for chemical risks to humans.

Results: We examined how dose-dependent responses of zebrafish embryos exposed to flusilazole can be extrapolated, using pathway point of departure data and reverse toxicokinetics, to obtain human oral dose hazard values that are similar to published mammalian chronic toxicity values for the chemical. We also examined how development/safety data for human health can be used to help assess potential risks of pharmaceuticals to nontarget species in the environment.

Discussion: Using several examples, we demonstrate that pathway-based analysis of chemical effects provides new opportunities to use alternative models (nonmammalian species, *in vitro* tests) to support decision making while reducing animal use and associated costs.

Conclusions: These analyses and examples demonstrate how alternative models can be used to reduce cost and animal use while being protective of both human and ecological health.

Citation: Perkins EJ, Ankley GT, Crofton KM, Garcia-Reyero N, LaLone CA, Johnson MS, Tietge JE, Villeneuve DL. 2013. Current perspectives on the use of alternative species in human health and ecological hazard assessments. Environ Health Perspect 121:1002–1010; http://dx.doi.org/10.1289/ehp.1306638

## Introduction

The use of traditional animal models and assays to assess the potential human and ecological hazards and risks posed by tens of thousands of chemicals that are currently being evaluated both in Europe and the United States would be prohibitively costly and time consuming, and vastly increase the number of testing animals needed (Rovida and Hartung 2009). As a result, toxicology has shifted from standard empirical testing to a pathway-based vision relying on *in vitro* systems and predictive models [National Research Council (NRC) 2007]. Although the challenges differ, a pathway-based vision is equally applicable to ecotoxicology (Villeneuve and Garcia-Reyero 2010).

As regulatory toxicology increases its reliance on predictive approaches, the historical distinction between human and ecological toxicology is increasingly blurred. These disciplines should no longer be defined by the animal models they employ. Greater emphasis should be placed on understanding chemical perturbation(s) of pathways at key junctures, including activation or inactivation of specific receptors, enzymes, or transport proteins (molecular initiating events) that in many instances are conserved across species.

Our increasing knowledge of pathway conservation facilitates the use of nontraditional species as toxicological models. Extrapolation across species, and selection of testing organisms, can be improved by focusing on the similarity (or lack thereof) of biological pathways among species, as opposed to direct comparisons of apical responses alone via species sensitivity distributions. Fundamental pathways underlying development (Adamska et al. 2007; Martindale 2005; Vallee et al. 2008), reproduction (Ankley and Johnson 2004), and stress response (Simmons et al. 2009) are highly conserved in metazoans. Nonmammalian models such as zebrafish have been found to possess orthologs for 62% of all human genes (Howe et al. 2013). Species as phylogenetically remote as *Drosophila* possess pathways important in human disease and development such as, for example, the lateral inhibition pathway involved in lung cancer and sleep regulation (Chen et al. 1997; Foltenyi et al. 2007). Numerous studies have identified conserved pathways for diseases in nonmammalian vertebrates and invertebrates, further supporting the use of alternative models for toxicity testing (Embry et al. 2010; Hill 2005). Although caution is still needed in extrapolation, the similarities between humans and nontraditional species provide great potential for improving efficiencies in hazard assessments.

Embryos offer alternatives to *in vivo* testing with adult animals, but embryos may not have a complete organ system (e.g., reproductive) or metabolic capacity (Embry et al. 2010). Nevertheless, transcriptional analysis of zebrafish embryos exposed to ethinylestradiol or genistein have detected alterations to genes and pathways involved in estradiol response, steroid biosynthesis, and neurodevelopment, demonstrating that the developing embryo has potential in screening for endocrine-disrupting chemicals that affect reproduction (Schiller et al. 2013; Vosges et al. 2010). Zebrafish embryos can also be predictive of *in vivo* chemical effects in both adult fish and rats, depending on the pathway involved. Knöbel et al. (2012) found that chemical toxicity to zebrafish embryos was predictive of acute toxicity in adult fish, with the possible exception of chemicals requiring metabolic activation. Enough pathway conservation is present in 24 hr post-fertilized zebrafish embryos that the toxicity of 60 chemicals was well correlated to toxicity in rats (Ali et al. 2011). This correlation was also dependent on the chemical class examined where carboxylic acids, glycosides, and alkaloids were more toxic to zebrafish, whereas alcohols were more toxic to rats than zebrafish. Zebrafish embryos also have complete pathways for thyroid hormone synthesis (Thienpont et al. 2011), heart development and more (Hill 2005). While it remains to be shown that embryo tests are fully predictive of effects in other species, evidence to date supports the view that fish embryos can be protective of both adult alternative species and mammals when used in a pathway context.

Here we postulate that, using a pathway-based hazard assessment approach, data from multiple species and non-animal alternative models are equally valuable for both ecological and human health hazard assessment. Using the adverse outcome pathway (AOP) framework (Ankley et al. 2010), we provide examples of how data from human-focused assays can be useful in identifying key initiators and predicting effects in nonmammalian species. Likewise, we describe how alternative models can be predictive of effects of human health concern (e.g., endocrine disruption) and link chemicals to toxicity pathways, or modes of action, in both mammals and ecological species. Finally, we demonstrate how dose-dependent effects in alternative models can be translated using a pathway-based measure to chemical hazard levels that are similar to those generated using mammalian species in chronic tests. These examples highlight the scientifically credible foundation that supports the predictive application and/or extrapolation of pathway-based toxicological data across species.

## Use of Alternative Species and *in Vitro* Assays in an AOP Framework

A mechanistic understanding of the effects of pathway perturbation is required to accurately relate chemical impacts across species. Adverse outcome pathways provide a framework that organizes mechanistic and/or predictive relationships between initial chemical–biological interactions (i.e., molecular initiating events, or MIEs), pathways, and adverse phenotypic outcomes relevant to hazard assessment. Using an AOP framework allows the use of alternative models by informing extrapolation of chemical impacts across species.

Extrapolation across species may occur at several different levels once an AOP, or even elements of one, has been defined. The simplest is at the molecular initiating event level where sequences or structures of proteins can be compared. More complex extrapolations occur at a pathway level, from molecular initiating event to adverse outcome including the sequence of events and the dose or threshold concentrations required to activate these events. Pathways can be explored either as discrete pathways or as networks using cross-species comparative genomics. [For a brief review, see Burgess-Herbert and Euling (2011).] Ultimately, the most realistic approach is to translate effects through systems-level models where dynamic events are incorporated, such as chemical concentrations, homeostasis, effects over time, and species-specific parameters related to absorption, distribution, metabolism, and excretion.

*Extrapolation at the molecular initiating event level facilitates prediction of potential adverse effects.* Cross-species comparisons focused on conserved events permit anchoring an AOP in a nonmammalian species to those relevant to rodents or humans through the identification of common biological machinery, under the assumption that evolutionarily conserved proteins may have conserved functions. Identification of a protein ortholog for a known chemical molecular target can be used to infer possible effects, especially where an AOP exists. Numerous human drug targets are conserved across ecologically relevant vertebrate and nonvertebrate species (Gunnarsson et al. 2008; Huggett et al. 2003; Kostich and Lazorchak 2008). Assigning functions via sequence similarity should be done with caution because genome duplication in species such as zebrafish has resulted in multiple orthologs for 15% of human genes (Howe et al. 2013). Duplicated genes are generally free to evolve and acquire new functions, which can confuse functional attribution by sequence similarity.

Recent concerns regarding the potential of pharmaceuticals to harm nontarget aquatic organisms illustrate how extrapolation of human-focused data can be used to infer potential ecological effects for prioritizing pharmaceutical chemicals/classes for hazard assessment purposes (Boxall et al. 2012; Schreiber et al. 2011; Tarazona et al. 2007). For example, although the estrogen receptor (ER) is well conserved across vertebrate species (Baker 2011), a functional ortholog has not been found in invertebrate species (Baker et al. 2008; Thomson et al. 2009). Therefore, chemicals that bind to the ER should affect vertebrates more than invertebrates, regardless of dose—a prediction confirmed in comparative studies (Goto and Hiromi 2003; Jukosky et al. 2008; Santos et al. 2007).

*Using human-focused molecular initiating event assays to predict higher-level effects across species.* Most, if not all high throughput screening (HTS) programs designed to assess the toxicity or biological effects of chemicals are centered on molecular initiating events known to be relevant to human health (Collins et al. 2008). Where these events are conserved across species, the HTS data could be relevant to ecological hazard assessment (Kavlock et al. 2012). This is demonstrated by examination of 309 chemicals tested in the U.S. Environmental Protection Agency (EPA) ToxCast™ phase I project (Knudsen et al. 2011) and of 40 chemicals for which fathead minnow 21-day short-term reproduction assay data have been reported in the peer-reviewed literature. Both HTS and fathead minnow reproduction data exist for nine chemicals [atrazine, bisphenol A (BPA), fenarimol, fipronil, methoxychlor, prochloraz, propiconazole, prometon, and vinclozolin; Table 1]. In general, significant responses in HTS assays for each of the nine chemicals (Knudsen et al. 2011; see Supplemental Material, Figures S1–S9) were predictive of the response in the fathead minnow (Table 1) when the HTS assays were relevant to established AOPs involving fish reproduction (Ankley et al. 2010).

**Table 1:** Extrapolation of MIEs predicts adverse outcomes in other species: Comparison of human *in vitro* screening of nine chemicals for potential MIEs related reproductive toxicity (ToxCast^TM^ MIE) to *in vivo* effects of the same chemicals on reproductive end points in female fathead minnows.

	ToxCast	*In vivo*, female fathead minnows		
	MIE linked to reproductive toxicity in fish^*a*^	Plasma E2	Plasma Vtg	Cumulative fecundity
Prochloraz^*b*^	Inhibition of CYP19A1 and AR binding	Sig ↓, 0.3 mg/L	Sig ↓, 0.1 mg/L	Sig ↓, 0.1 mg/L
Propiconazole^*c*^	Inhibition of CYP19A1	Sig ↓, 0.5 mg/L	Sig ↓, 0.5 mg/L	Sig ↓, 0.5 mg/L
Bisphenol A^*d*^	Interacted with ER and also with the AR at higher concentrations	Sig ↓, 10 μg/L	Sig ↓, 10 μg/L
Fenarimol^*b*^	None	Sig ↓, 1.0 mg/L	Sig ↓, 1.0 mg/L	Sig ↓, 1.0 mg/L
Vinclozolin^*e*^	None	No effect	Sig ↓, 0.4 mg/L	Sig ↓, 0.1 mg/L
Fipronil^*f*^	None	No effect (≤ 1 mg/L)	No effect (≤ 1 mg/L)	No effect (≤ 1 mg/L)
Prometon^*g*^	None	No effect (≤ 5 μg/L)	No effect (≤ 5 μg/L)	No effect (≤ 5 μg/L)
Methoxychlor^*h*^	None	No effect (≤ 5 μg/L)	No effect (≤ 5 μg/L)	Sig ↓, 5 μg/L
Atrazine^*i,j*^	None	No effect (≤ 50 μg/L)^*j*^	No effect (≤ 50 μg/L)^*j*^	Sig ↓, 0.5 μg/L^*i*^
				No effect (≤ 50 μg/L)
Abbreviations: E2, estradiol; MIE, molecular initiating event; sig ↓, significantly decreased; Vtg, vitellogenin.^***a***^Indicates whether activity was observed in one or more ToxCast assays (Knudsen et al. 2011) corresponding to molecular initiating events previously associated with reproductive toxicity in fish (Ankley et al. 2010). Specific MIEs of interest included androgen receptor (AR) agonism, estrogen receptor (ER) antagonism, and inhibition of steroidogenic enzyme activities, particularly aromatase (CYP19A1). ^***b***^All effects are after 21 days of continuous exposure. See Ankley et al. (2005). ^***c***^From a 96-hr range finding study, all other data are from a 21-day exposure (Skolness et al. 2013). ^***d***^Measured after 96 hr continuous exposure (Villeneuve et al. 2012). ^***e***^Martinović et al. 2008. ^***f***^Bencic et al. 2013. ^***g***^Villeneuve et al. 2006. ^***h***^Ankley et al. 2001. ^***i***^Tillitt et al. 2010. ^***j***^Bringolf et al. 2004.				

Reproductive effects of BPA in female fish were consistent with the ER interactions identified in HTS assays, (Table 1; see also Supplemental Material, Figure S1). Likewise, the two chemicals identified as aromatase (CYP191A) inhibitors (prochloraz and propiconazole; see Supplemental Material, Figures S2 and S3) caused reproductive and endocrine effects in the fathead minnow (Table 1) consistent with aromatase inhibition. This was true even when multiple molecular targets, including many with lower AC_50_ (half-maximal activity concentration) values than those related to reproduction, were affected (see Supplemental Material, Figures S1–​S9), indicating that the most sensitive assay may not be driving the toxicity outcome. Interestingly, the three chemicals that had either no impact (fipronil and prometon; Table 1) or inconsistent impacts on reproduction (atrazine; Table 1) had the least activity in HTS assays (see Supplemental Material, Figures S4–S6). No ER activity was detected in HTS with methoxychlor, a chemical for which metabolites of the parent chemical are thought to be largely responsible for its endocrine activities (Bulger et al. 1978; see also Supplemental Material, Figure S7). However, an interaction with the androgen receptor (AR) was one of the few activities detected for vinclozolin, another chemical with endocrine-active metabolites (Kelce et al. 1994; see Supplemental Material, Figure S8). For only one of the nine chemicals (fenarimol) was there an apparent disagreement between the HTS results and fish reproduction results that was not readily explainable. *In vivo* results suggested ER antagonism as a mode of action (Ankley et al. 2005) (Table 1). However, HTS results did not identify interactions with the ER as a likely event (see Supplemental Material, Figure S9). The reason for the discrepancy remains unclear but raises the possibility that the hypothesized *in vivo* mode of action may be inaccurate.

Clearly, human- or mammal-focused HTS assays targeting conserved molecular initiating events can be useful in predicting potential higher-level effects in other species. The failure to identify a relevant molecular initiating event for methoxychlor in HTS assays demonstrates the current limitations of *in vitro* screening in predicting *in vivo* activities of chemicals that are metabolically activated. Nonetheless, currently available data supports the application of mammalian molecular screening data to assessing chemical hazards for a range of nonmammalian species, provided that relevant toxicity pathways are reasonably conserved and understood. Data on the binding kinetics of chemicals to receptors from multiple species, in addition to toxicokinetic and toxicodynamic information, will be necessary to begin quantitatively predicting effects across species using HTS data.

**Figure 1:**
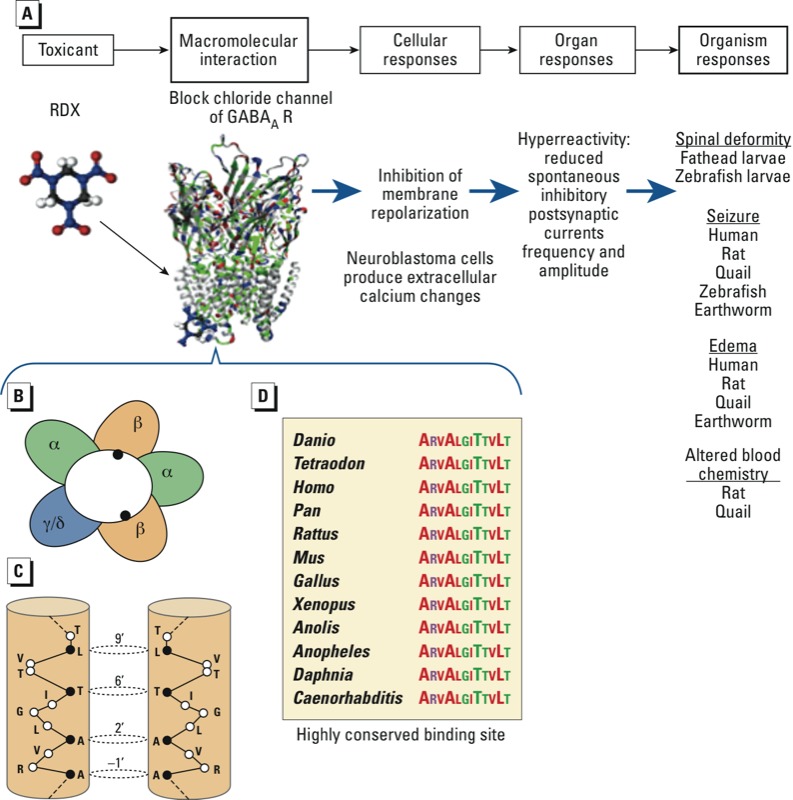
Cross-species similarity of GABA_A_ receptor (GABA_A_R) and RDX toxicity in an AOP framework. (*A*) Schematic of pathway. (*B*) Schematic view of a GABA_A_R heteropentamer with the channel in the center formed of two copies of an α subunit (α1–α6), two copies of a β subunit (β1, β2, or β3), and a third subunit of γ or δ protein (adapted from Olsen 2006). (*C*) Cytoplasmic end of the transmembrane channel for two β-3 subunits, indicated by closed circles in (*B*) in GABA_A_R, showing the residues that form the binding site for picrotoxin (PTX) and RDX [closed circles in (*C*): residues numbered 1’–23’ from the N-terminal bottom; open circles represent residues that are not part of the binding site]. (*D*) Sequence alignment of the PTX/RDX binding site for several species: *Danio rerio, Tetraodon nigroviridis, Homo sapiens, Pan troglodytes, Rattus norvegicus, Mus musculus, Gallus gallus, Xenopus tropicalis, Anolis carolinensis, Anopheles gambiae, Daphnia pulex, and Caenorhabditis elegans.*

*Pathway conservation also enables assessment of toxicity in alternative species based on mammalian AOP models.* Where orthologous pathways occur, pathway-based data can often be extended well beyond the phylogenetic group from which it was derived. For example, hexahydro-1,3,5-trinitro-1,3,5-trizine (RDX) causes seizures and other indicators of neurotoxicity in species as varied as human, rat, quail, and earthworm (Garcia-Reyero et al. 2011) (Figure 1). Williams et al. (2010) screened for RDX binding to different neurological receptors and found that RDX binds to the picrotoxin binding site in the chloride channel of the γ-aminobutyric acid A (GABA_A_) receptor, an interaction associated with the onset of seizures in rats. The picrotoxin binding site in the GABA_A_ receptor is highly conserved from humans to earthworms, indicating that binding the GABA_A_ receptor is a likely molecular initiating event in multiple species (Garcia-Reyero et al. 2011) (Figure 1). Given the conserved role of GABAergic signaling, results of assays assessing impacts on this pathway are likely to be informative of potential effects across a similarly broad range of species and can provide a reasonable basis for extrapolating mammalian health data to other species.

*Pathway conservation enables prediction of potential endocrine activity across species.* The hypothalamus–pituitary–gonad (HPG) axis is one example of a system highly conserved across vertebrates (Norris 2007). Conservation of molecular initiating events across species should enable nonmammalian vertebrate models to be used in determining the potential for chemicals to interact with the HPG axis in any vertebrate species (including humans), despite the fact that actual apical responses associated with endocrine disruption may differ across species.

Direct consideration of this hypothesis can be done using data from the U.S. EPA Endocrine Disruptor Screening Program (EDSP), which uses multiple *in vivo* tests with rats and fish as screening tests (Tier 1) to identify substances with the potential to interact with the estrogen, androgen, or thyroid hormone system which are then characterized in depth using Tier 2 *in vivo* assays (Marty et al. 2011). The rat tests include *a*) the 3-day uterotrophic assay, which detects ER agonists through increases in uterine weight; *b*) the 10-day Hershberger assay, which detects AR agonists or antagonists through changes in weights of several androgen-responsive tissues; and *c*) two pubertal assays with males and females, 20–30 days in length, which detect impacts on ER- and AR-mediated responses, including sex steroid synthesis. The EDSP Tier 1 fish test is a 21-day fathead minnow reproduction assay featuring a range of biochemical, histological, and apical end points that capture chemical effects on multiple HPG pathways, including activation and antagonism of ERs and ARsas well as inhibition of sex steroid synthesis (Ankley et al. 2001). Because fecundity is a general indicator of reproduction, the fish assay can detect a broader range of chemical effects including those acting via non-endocrine mechanisms.

Ankley and Gray (2013) examined results from method validation studies with 12 model endocrine-active chemicals, which act via several different molecular initiating events, that were tested both in the fathead minnow and one or more of the rat EDSP assays; they confirmed that effects on a highly conserved toxicological pathway in one species are predictive of effects in the other species (Table 2). For example, both species were responsive to ethinylestradiol and methoxychlor, which are known to activate ER-mediated pathways, although estrogenic activity in fish was manifested as induction of vitellogenin and in rats as changes to relative weights of estrogen-responsive tissues (uterotrophic and female pubertal assays). Responses of the fish and female rat EDSP tests to a third weak ER agonist (BPA) were more variable, demonstrating a lesser effect in the rats that was likely due to first-pass hepatic metabolism associated with oral dosing and highlighting the fact that metabolism and route of exposure need to be considered in cross-species extrapolation, in addition to pathway conservation.

**Table 2:** Conservation of endocrine-active chemicals effects in rats and fish (fathead minnow).

Pathway	Chemical	Assay				
		Rat uterotrophic	Rat Hershberger	Rat pubertal female	Rat pubertal male	Fathead minnow
Estrogen agonist	17α‑Ethynylestradiol	+	NT	+	NT	+
	Methoxychlor	+	–	+	–/+	+
	Bisphenol A	+	–	–	NT	+
Androgen agonist	Methyltestosterone	+	+	NT	+	+
	17β-Trenbolone	NT	+	NT	NT	+
Androgen antagonist	Flutamide	NT	+	NT	+	+
	Vinclozolin	NT	+	NT	+	+
	*p, p*´-DDE	–	+	NT	+	+
Steroidogenesis	Ketoconazole	NT	NT	+	+	+
	Fadrozole	NT	NT	+	NT	+
	Fenarimol	NT	NT	–	NT	+
	Prochloraz	+	NT	NT	+	+
Abbreviations: NT, not tested. +, positive result. –, negative result. An overview of comparative responses of the various U.S. EPA Endocrine Disruptor Screening Program Tier 1 *in vivo* assays to chemicals representing different pathways within the vertebrate HPG axis. Modified from Ankley and Grey (2013).						

Similar results were seen in rodent and fish assays for AR agonists and antagonists [methyltestosterone, 17β-trenbolone, 1,1-dichloro-2,2-bis(*p*-chlorophenyl) ethylene (*p,p*´-DDE), flutamide, and vinclozolin; Table 2]. That is, although the apical end points that were affected differed, changes in the end points in the two species reflected the conserved molecular initiating event. Chemicals inhibiting sex steroid synthesis (ketoconazole, fadrozole, and prochloraz) were also consistently detected in both the fish assay and one or more rat assays (Table 2). A fourth sex steroid synthesis inhibitor (fenarimol) was detected in the fish assay but not with *in vivo* rat assays (Table 2). Although not detected with *in vivo* tests, fenarimol has been identified as a sex steroid synthesis inhibitor using the mammal-based H295R *in vitro* assay (Villeneuve et al. 2007). In the context of the 12 model chemicals examined, the fathead minnow assays were essentially predictive of pathway-specific responses in the four rodent assays. The Tier 1 tests were focused on sensitively detecting the potential to cause effects, rather than accurate quantification of hazard thresholds. Therefore, the ability of nonmammalian models to quantitatively predict hazard effects in mammals requires further examination and development. This analysis demonstrates that apical effects can be predicted across species if conserved molecular initiating event and pathways exist and, as such, supports hazard screening and assessment for HPG-active chemicals applicable both to mammalian and nonmammalian (vertebrate) species.

*Pathway conservation enables effective resource use in prediction of thyroid and developmental effects on both mammalian and nonmammalian species.* Pathways associated with the hypothalamic–pituitary–thyroidal (HPT) axis are common and important features of embryonic development and metabolic control in all vertebrate species (Paris and Laudet 2008). Early development in vertebrates is typically characterized by transient elevations of thyroid hormone (TH) that elicit species-specific physiological and morphogenetic responses with lasting developmental consequences as seen in the metamorphosis of free-swimming tadpoles into juveniles (Dodd 1976; Leloup and Buscaglia 1977), reorganization of the flatfish body (Power et al. 2001), and a TH-dependent shift in physiology in salmonids during migration (Dickhoff et al. 1978). Further, vertebrate post-embryonic neurodevelopment is TH-dependent in that deviations from normal TH concentrations can result in neurological defects and deficits (Zoeller and Rovet 2004). As a result, chemical disruption of TH-dependent pathways in vertebrates can have significant adverse impacts (Zoeller 2007).

**Figure 2:**
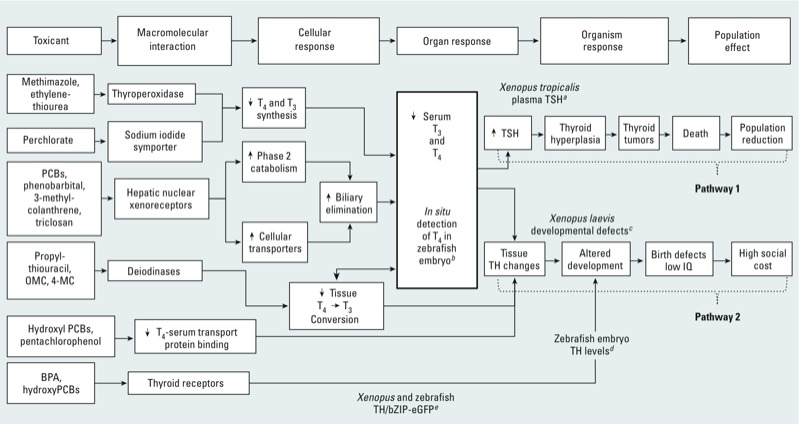
Major AOPs for thyroid disruption with example toxicants and alternative models applicable to both human and ecological hazard assessment. The thick black outlined box indicates the critical event of serum level concentrations of thyroid hormones. Pathway 1: rat pathway leading to tumors via thyroid hyperplasia. Pathway 2: principle pathway of concern affecting humans. Abbreviations: IQ, intelligence quotient; 4-MC, 4-methylbenzylidene camphor; OMC, octyl methoxycinnamate; T_3_, triiodothyronine; T_4_, thyroxine; TR, thyroid receptor. Figure modified from Crofton (2008). ^*a*^Quantification of plasma TSH levels in *Xenopus tropicalis* (Korte et al 2011). ^*b*^Direct quantification of intrafollicular concentrations of T_4_ in zebra­fish embryos (Thienpont et al. 2011). ^*c*^Detection of developmental defects with *X. laevis* metamorphosis assay (Degitz et al. 2005 Organisation for Economic Co-operation and Development 2004). ^*d*^Detection of developmental defects using zebrafish embryos. ^*e*^Reporter gene (eGFP) detection of TR activity (Fini et al. 2007).

Disruption of TH activity can result from several established events (Crofton 2008; Figure 2). Of these, thyroid peroxidase and sodium iodide symporter are key proteins in the metabolic pathway of TH. Both thyroid peroxidase and sodium iodide symporters from numerous species are inhibited by the same chemicals resulting in readily predictable reductions in circulating TH, though downstream effects may be species specific. Other molecular initiating events affecting TH activity (i.e., enhanced phase II metabolism of THs via glucuronosyl- or sulfo-transferases, enhanced cellular transport of thyroid hormone, deiodinase inhibition, and interference with thyroid receptor function) are found primarily in peripheral tissues where chemical effects and subsequent consequences are highly variable among species, as is the effectiveness of cross-species extrapolation using these mechanisms. For example, these other events can lead to thyroid hypertrophy, hyperplasia, and thyroid follicular tumors in rats, which are not relevant to the mode of action of concern for humans (Capen 1997; Hill et al. 1998) (Figure 2, pathway 1). Conversely, in frogs, these same events can lead to decreased serum TH, decreased tissue TH, decreased tissue mRNA and protein synthesis, and disruption of development [which is relevant to the mode of action of concern for humans (Degitz et al. 2005) (Figure 2, pathway 2)].

Rodent studies have long been a standard way to assess the impact of chemicals on thyroid function (Capen 1997). Alternative species are useful in evaluating the risks, both to humans and other vertebrates, associated with chemicals that perturb the HPT axis. The African clawed frog, *Xenopus laevis*, is a well-documented developmental and physiological model used to assess thyroid disruption (Degitz et al. 2005; Miyata and Ose 2012; Pickford 2010). A comparison of amphibian and mammalian models for detecting thyroid disruption found good concordance in that both detected the majority of 32 environmental chemicals (Pickford 2010). An advantage of the assays using *X. laevis,* when compared with mammals, is that effects on thyroid disruption can be observed via distinct, relatively specific changes in development (i.e., metamorphosis) as opposed to the more subtle changes in humans and other mammals, such as behavioral and neural developmental effects requiring extensive resources to assess (Grim et al. 2009; Nieuwkoop and Faber 1994). An additional advantage of the amphibian model is that there is no serum protein sink/buffer to bind TH as is found in rodents to complicate the toxicokinetics of TH responses.

Like other vertebrates, *X. laevis* responds to low levels of TH by release of thyroid stimulating hormone (TSH) from the pituitary to upregulate TH synthesis and release by the thyroid gland through a negative feedback loop. Adverse outcomes occur when the degree of inhibition of TH synthesis and release exceeds the ability of the feedback loop to maintain TH levels. As a result, serum TH levels are a reliable predictor of HPT axis disruption across species. Methods to measure circulating TSH in *X. laevis* have been recently developed and successfully applied in a toxicological context (Korte et al. 2011; Tietge et al. 2012). Zebrafish embryos have also been used to directly quantify thyroid disruption effects through immunodetection of TH levels in intrafollicular cells (Thienpont et al. 2011). Receptor function can be examined *in vivo* through use of transgenic *X. laevis* thyroid receptor-activated promoter reporter systems (Terrien et al. 2011). These alternative species models clearly provide data relevant to both human and ecological risk assessment when analyzed in a pathway context, yet allowing refinement for follow-on studies if needed (Figure 2).

*Use of alternative species as models to define and refine adverse effects of chemicals.* Alternative species provide models that can be manipulated to assess effects on subtle outcomes such as motor and sensory behaviors and cognitive function (Levin and Tanguay 2011; MacPhail et al. 2009), validate *in vivo* predictions and provide useful *in vivo* data for human health hazard assessment. Alternative models are especially useful in understanding the specific mechanisms and pathways through which chemicals cause toxicity to mammals, thereby reducing and refining the use of mammalian models. For example, thalidomide has been identified as a prenatal developmental toxicant and vascular disrupting compound based on potential molecular initiating events identified through HTS results (Kleinstreuer et al. 2011; Sipes et al. 2011). Although thalidomide is a well known developmental toxicant and teratogen in humans, *in vitro* studies in zebrafish embryos have helped define that it causes limb malformation and other developmental defects through the thalidomide-binding protein cereblon, a protein important in limb outgrowth, and the fibroblast growth factor, Fgf8, which disrupts vascular development (D’Amato et al. 1994; Ito et al. 2010; Yabu et al. 2005).

Perfluorooctane sulfonate (PFOS), a breakdown product of perfluorinated surfactants, has also been identified as a developmental toxicant and vascular disrupting compound via HTS. Effects seen in alternative models are consistent with this prediction and also predictive of effects observed in mammals, including humans. PFOS has been shown to impair cardiac development in marine medaka embryos (Huang et al. 2011); cause altered cardiac function, behavior, and developmental toxicity in zebrafish embryos (Huang et al. 2010); and alter lipid metabolism in salmon larvae (Arukwe et al. 2013). In mammals, PFOS has been shown to cause developmental, reproductive, neuroendocrine, and hepatic steatosis effects (Austin et al. 2003; Bijland et al. 2011; Thibodeaux et al. 2003). PFOS has also been linked to altered cholesterol levels in human epidemiological studies (Eriksen et al. 2013). In addition, demonstration of thyroid disruption by environmentally relevant levels of PFOS in *X. laevis* (Cheng et al. 2011) confirms early observations that PFOS may impact thyroid function in humans (Organisation for Economic Co-operation and Development 2002). Used in a pathway context, nonmammalian vertebrates and embryo tests can clearly show similar effects to chemicals as found in mammals.

*Extrapolation of dose–response relationships between species for hazard assessment.* Alternative models offer significant advantages to answering questions requiring a well-characterized constant, controlled exposure to test chemicals where low-dose and compensatory effects are important considerations. For example, Ankley et al. (2009) conducted several intensive studies to characterize dose/time–dependent changes in the fathead minnow HPG function relative to direct effects, compensation, and recovery to collect robust data sets with *a*) temporally intensive sampling of several hundred animals over a short period, and *b*) use of controlled, aqueous exposures for highly consistent and predictable dosimetry (Ankley et al. 2009). The aqueous exposures used in fish tests provide constant exposure levels not found in the oral and dermal exposure methods commonly used in mammalian studies that result in fluctuating internal doses rather than a truly constant exposure. So, although relevant to real-world exposures in human health, oral and dermal exposure routes complicate understanding system dynamics because of the complex interplay between biology (e.g., signaling, feedback) and varying chemical intensity.

Used in a pathway context, nonmammalian vertebrates and embryo tests can show effects similar to those found in mammals; however, the concentrations needed to cause an effect and the mechanisms of compensation may be different. Both commonalities and differences underlying response effects can be extrapolated between species using transcriptomics and proteomics to identify genes and signaling pathways in one species that can then be mapped to functional pathways that are conserved across species (Bauer-Mehren et al. 2009). Concentrations required to activate pathways and pathway data can be linked to adverse effects either by known functional linkages (key signaling cascades, metabolism, or experimental demonstration) or *de novo* linkages (data driven approaches such as network analysis or functional genomics with gene knockout and rescue experiments). Mapping of concentration-responsive genes to pathways would allow use of pathway and concentration data in more traditional risk frameworks that use no observable effect level (NOEL), no observed adverse effect level (NOAEL), or benchmark concentration methods to calculate a point of departure of a biomarker or phenotype related to an adverse effect in an exposed animal group from that of a control animal group. Because regulatory agencies use such a point of departure, modified by uncertainty factors, to set safe levels for chemical exposures (Gaylor and Aylward 2004), dose–response effects could potentially be translated into values more amenable to current risk or hazard frameworks.

Choosing a key event in a pathway as a point of departure is currently difficult (Miller et al. 2009; Woodruff et al. 2008). An alternative is to use a pathway-based point of departure derived from the lowest concentration at which a functionally enriched, co-regulated cluster of transcripts is significantly different from controls. Ideally this pathway would be significantly linked to an adverse effect so that pathways related to compensation or secondary effects are not mistaken for toxicity. However, even changes proceeding from, or indirectly related to, adverse effects may be indicators of sensitivity to a chemical and therefore useful in hazard assessment and prioritization (Thomas et al. 2011). For example, treatment of zebrafish embryos from 0 to 24 hr after fertilization with flusilazole, a known developmental toxicant, caused a concentration-dependent response in morphological effects (Hermsen et al. 2012). Developmental delays, pericardial edema, and malformations of head and heart were observed at concentrations > 28 µM, whereas 2.8 µM flusilazole was the lowest concentration where functionally enriched, co-regulated clusters were observed. Retinol metabolism, a pathway highly conserved across vertebrates, was the most significant function changed at 2.8 µM. Because deregulation of this pathway is linked to developmental skeletal deformities and the mechanism is conserved in both zebrafish and mammals (Laue et al. 2011), a pathway-based NOEL of 1.35 µM and a lowest observable effect level of 2.8 µM flusilazole could be reasonably derived from the point at which the retinol metabolism pathway significantly departed from the control. Use of key events upstream as conservative points of departure has been widely used in mammalian toxicology (Hill et al. 1998; NRC 2005).

A disadvantage to extrapolation based on experimental data is that it is limited to the range of concentrations or doses used. As an alternative, benchmark dose or concentration modeling, which estimates the point at which chemically treated groups diverge from a control group by regression modeling of response curves, can be used to identify low-concentration effects below those concentrations empirically tested (Crump 1995). This approach has been applied to toxicogenomic- and HTS-based pathway data to derive quantitative hazard values from short-term *in vivo* rat exposures and human-focused *in vitro* assays (Burgoon and Zacharewski 2008; Judson et al. 2011; Thomas et al. 2011). When applied to toxicogenomic data from flusilazole-exposed zebrafish embryos, benchmark concentration modeling identified retinol metabolism as the most sensitive pathway, with a pathway-based benchmark concentration no effect level much lower than the pathway-based NOEL and closer to observed values in adult fish and values predicted by ToxCast™ analyses (Table 3). This data could potentially be used as a threshold level of sensitivity or as a no observable pathway effect level that could be extrapolated across species using species-specific toxicokinetic and toxicodynamic modeling.

**Table 3:** Dose– and concentration–response values can be compared across species and *in vitro* models using pathway-based measures and reverse toxicokinetics to derive a common hazard value for prioritization.

Animal model	Dose/concentration reference point	Toxicity value	Human lower oral equivalent (mg/kg/day)
Dog	Chronic oral, NOEL	0.20 mg/kg/day^*a*^	0.002^*a*^
Zebrafish embryo	24 hr, pathway NOEL	1.35 μM^*b*^
Zebrafish embryo	24 hr, pathway BMCL	0.310 μM	0.037
ToxCast™ *in vitro*	Most sensitive assay, BPAD	0.023 μM	0.003^*c*^
Fathead minnow	252-day flow through, NOEL	0.073 μM^*d*^	0.009
Rainbow trout	96-hr acute toxicity, NOEL	0.010 μM^*e*^	0.001
No observable effect levels (NOEL), biological pathway altering dose (BPAD), and pathway-based bench mark concentration lower level (pathway BMCL) values from flusilazole exposures in animal and *in vitro* models were converted to human oral 95% lower bound dose equivalents using human reverse toxicokinetics (Wetmore et al. 2012). The pathway BMCL for zebrafish was derived from gene expression data (Thomas et al. 2011).^***a***^U.S. EPA 2007. ^***b***^Hermsen et al. 2012. ^***c***^Wetmore et al. 2012. ^***d***^European Commission 2007. ^***e***^Food and Agricultural Organization of the United Nations 2008.			

*Determination of species-specific dose and kinetic parameters.* Toxicokinetic and toxicodynamic modeling is an important step in extrapolating from pathway- or effects-based concentration–response values to whole-animal chemical hazards. The absorption, distribution, metabolism, and elimination of chemicals have been studied extensively in humans and, increasingly, in alternative models. Huggett et al. (2003) predicted pharmacological responses in fish utilizing human therapeutic plasma concentrations [measured maximum concentration (C_max_)] normalized to predicted steady-state fish plasma concentrations to determine whether further toxicity testing may be warranted. In another study, Berninger and Brooks (2010) found that the ratio of acutely toxic drug dose to therapeutic drug dose in mammals has the potential to be predictive of chronic responses to pharmaceuticals in fish. Other methods, such as the well-stirred liver homogenate model, have been useful in predicting hepatic clearance measures of a chemical in trout as a means to estimate metabolism and bioaccumulation (Han et al. 2007). A logical next step will be to determine whether readily available human toxicokinetic–toxicodynamic information can be predictive of the action of a chemical in select aquatic species, or vice versa.

Species-specific toxicokinetic–toxicodynamic modeling, pathway-based point of departures, and modeling of uncertainty and population variability can be combined to translate dose responses in one species to NOEL-like dose values for another species. Judson et al. (2011) used the concept of biological pathway–altering concentration as a measure of the lowest chemical concentration at which an *in vitro* assay in a pathway is significantly changed. Once defined, the biological pathway–altering concentration was then extrapolated using reverse toxicokinetic modeling to estimate the external dose required to achieve the internal dose that is equal to the biological pathway–altering concentration, ultimately yielding a human dose equivalent that is required to cause toxicity. A reverse toxicokinetics model, suitable for chemicals that are mainly eliminated through metabolism and renal excretion, can be used to obtain a concentration-to-dose scaling factor in micromoles per milligram per kilogram body weight per day (Wetmore et al. 2012). The advantage of this approach is that simple assays exist for the rate of disappearance of parent chemical via hepatic metabolism and the fraction of chemical bound (or conversely unbound) to plasma proteins and allows one to tailor these parameters to specific species, including sensitive ecological species or humans (Han et al. 2007; Pacifici and Viani 1992).

Quantitatively, extrapolation across species can be illustrated by applying pathway-based benchmark concentration modeling in combination with the human reverse toxicokinetic modeling of Wetmore et al. (2012) to the flusilazole concentration-responsive retinol metabolism pathway of zebrafish embryos, described above. When the pathway-based benchmark concentration lower limit for flusilazole and zebrafish embryos is extrapolated using the human dose scaling factor determined for flusilazole (Wetmore et al. 2012), an oral dose equivalent required to cause an effect in humans is derived. In the case of flusilazole, values extrapolated from zebrafish embryos are within an order of magnitude of no effect level values derived from mammalian data and high throughput chemical hazard assessment values for prioritization derived from ToxCast™ assays (Table 3). Reverse toxicokinetics can also be used to extrapolate no effect levels from acute and chronic adult fish tests yielding values similar to those from *in vitro* assays and mammalian data (Table 3). These observations are consistent with a high conservation of flusilazole’s mechanism of action, including concentration sensitivity, among vertebrate species, such that effects seen in one species can be predictive of effects on another perhaps even to the extent of quantitatively identifying chemical hazard levels.

## Conclusions

As we move from largely empirical approaches in chemical safety assessment toward a more predictive paradigm focusing on perturbation of well-conserved pathways and processes, toxicologists should have greater flexibility in the selection of model organisms for testing. As part of this review, we have cited a body of literature documenting the effective use of nonmammalian vertebrates and invertebrates for studying mechanisms and pathway perturbations relevant to human disease. Using the AOP context, we provide evidence for the suitability and practicality of such models for applications such as neurotoxicology and for identifying endocrine-active chemicals, identifying AOPs, and extrapolating concentration–response effects to mammals. In turn, we illustrate how rich sources of human-oriented effects data can be efficiently employed to address pertinent ecological risk challenges such as prioritizing pharmaceutical contaminants in terms of potential effects in nontarget species. For both types of hazard assessments, data can be efficiently employed to address pertinent ecological risk challenges, such as prioritizing data from “alternative species.”

The use of alternative species and pathway-based approaches for hazard assessment is still evolving; therefore, caution should be used when applying these approaches so that ample scientific evidence is presented to support extrapolations and other conclusions. In some cases such as pathway-based benchmark concentrations, we do not yet understand the relationship between the chemically most sensitive pathway and a specific toxicological outcome. In other cases, rapidly evolving technologies such as next generation sequencing or biological network analysis do not have firmly established, reliable, and accurate analysis methods or standards. Although each approach and alternative model is likely to have limitations, the availability of numerous options at different levels of biological organization should compensate and enable more accurate assessment while using fewer animals.

In summary, the movement of toxicology and hazard assessment toward a pathway-based paradigm opens numerous opportunities in applying nontraditional approaches for hazard screening and understanding the risks of chemical exposures. Alternative species can provide valuable and relevant information more rapidly, and at lower cost, than traditional species used for human chemical risk assessment. In a pathway centric world, all species can provide information to protect other species, each with different advantages in use, sensitivity, and accessibility. Given this perspective, the distinctions between the disciplines of human health and ecotoxicology are blurring and heading towards a more unified and integrated application of toxicological data.

## Supplemental Material

(401 KB) PDF
