# Supplementary material for: Current Perspectives on the Use of Alternative Species in Human Health and Ecological Hazard Assessments
Source: Environ Health Perspect. 2013 Jun 14;121(9):1002–10. doi: 10.1289/ehp.1306638 (PMC3764090; doi:10.1289/ehp.1306638)
Supplement: (401 KB) PDF [file ehp.1306638.s001.pdf]

Environmental Health Perspectives doi:10.1289/ehp.1306638

Supplemental Material

Current Perspectives on the Use of Alternative Species in Human Health and Ecological Hazard Assessments

Edward J. Perkins, Gerald T. Ankley, Kevin M. Crofton, Natàlia Garcia-Reyero, Carlie A. LaLone, Mark S. Johnson, Joseph E. Tietge, and Daniel L. Villeneuve

Table of Contents

Figure S1. Results for bisphenol A tested in the ToxCast^{TM} battery of assays p 3

Figure S2. Results for prochloraz tested in the ToxCast^{TM} battery of assays p 4

Figure S3. Results for propiconazole A tested in the ToxCast^{TM} battery of assays p 5

Figure S4. Results for fipronil tested in the ToxCast^{TM} battery of assays p 6

Figure S5. Results for prometon tested in the ToxCast^{TM} battery of assays p 7

Figure S6. Results for atrazine tested in the ToxCast^{TM} battery of assays p 8

Figure S7. Results for methoxychlor tested in the ToxCast^{TM} battery of assays p 9

Figure S8. Results for vinclozolin tested in the ToxCast^{TM} battery of assays p 10

Figure S9. Results for fenarimol tested in the ToxCast^{TM} battery of assays p 11

Reference. p 12

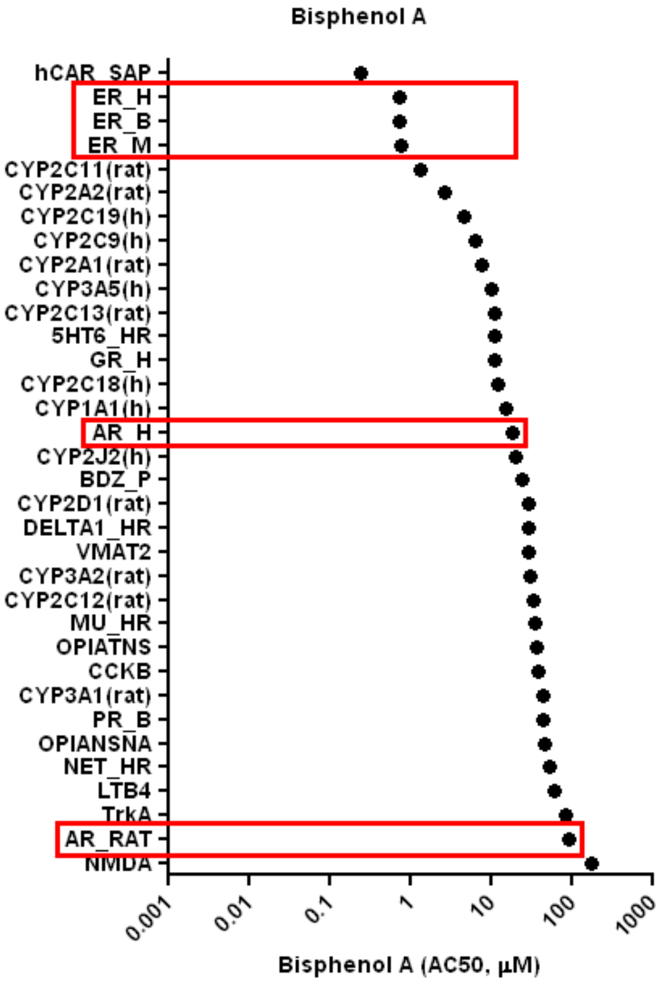

Supplemental Material, Figure S1. Molecular screening assay results for bisphenol A tested in the ToxCast™ battery of assays (Derived from Knudsen et al. 2011). An AC50 is the *in vitro* chemical concentration yielding 50% of the maximal activity produced by an assay-specific standard chemical. Assays were identified in which a significant response was detected. Assays were then ranked by AC50. Assays relevant to molecular initiating events associated with established reproductive adverse outcome pathways of fathead minnow (*Pimephales promelas*) are outlined in red.

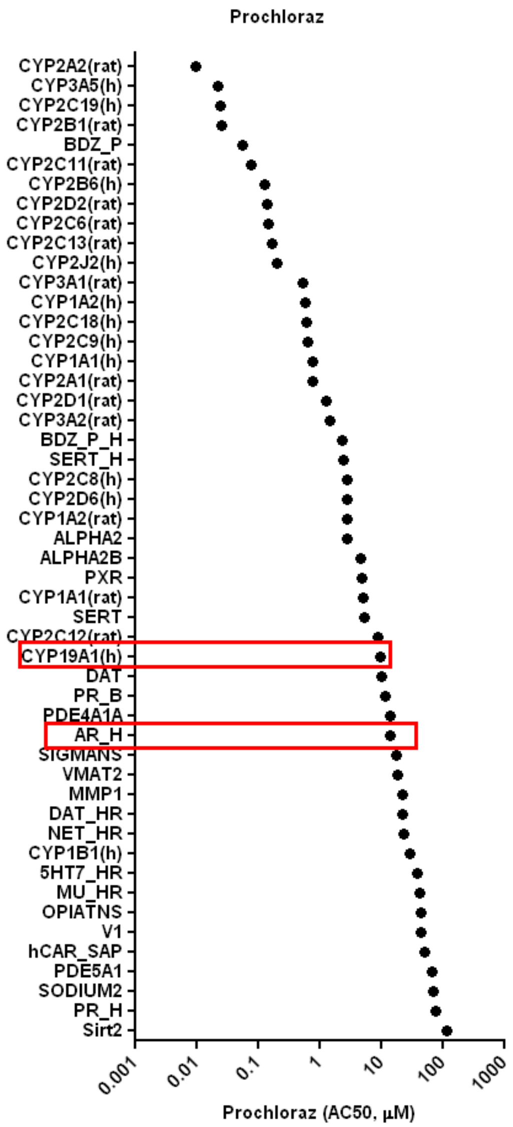

Supplemental Material, Figure S2. Molecular screening assay results for prochloraz tested in the ToxCast™ battery of assays (Derived from Knudsen et al. 2011). An AC50 is the *in vitro* chemical concentration yielding 50% of the maximal activity produced by an assay-specific standard chemical. Assays were identified in which a significant response was detected. Assays were then ranked by AC50. Assays relevant to molecular initiating events associated with established reproductive adverse outcome pathways of fathead minnow (*Pimephales promelas*) are outlined in red.

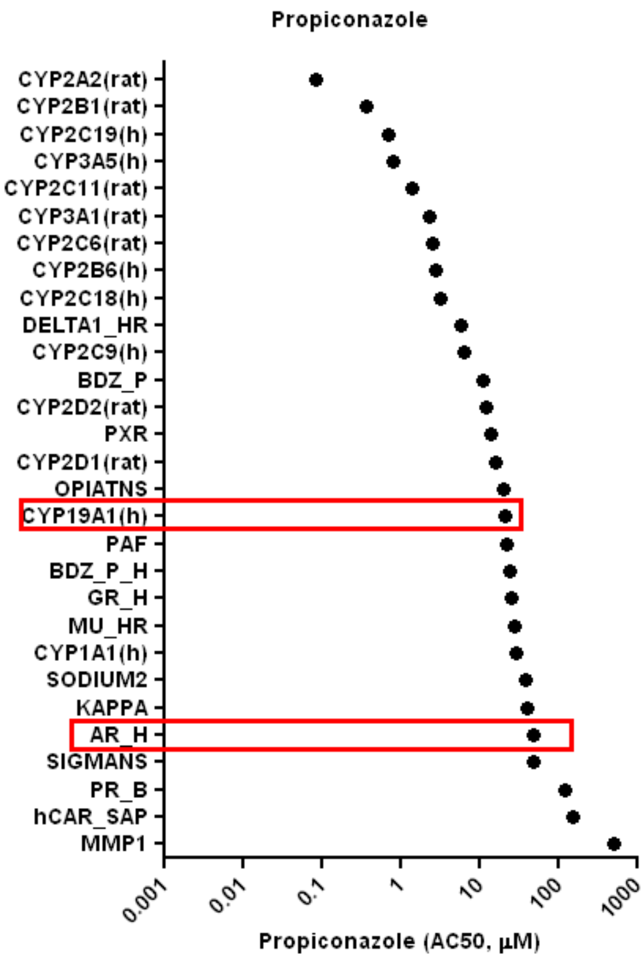

Supplemental Material, Figure S3. Molecular screening assay results for propiconazole tested in the ToxCast^{TM} battery of assays (Derived from Knudsen et al. 2011). An AC50 is the *in vitro* chemical concentration yielding 50% of the maximal activity produced by an assay-specific standard chemical. Assays were identified in which a significant response was detected. Assays were then ranked by AC50. Assays relevant to molecular initiating events associated with established reproductive adverse outcome pathways of fathead minnow (*Pimephales promelas*) are outlined in red.

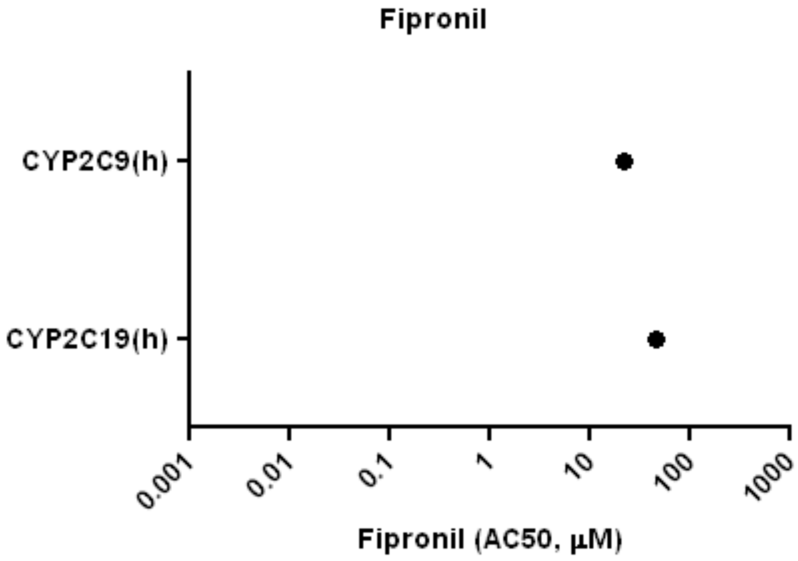

Supplemental Material, Figure S4. Molecular screening assay results for fipronil tested in the ToxCast^{TM} battery of assays (Derived from Knudsen et al. 2011). An AC50 is the *in vitro* chemical concentration yielding 50% of the maximal activity produced by an assay-specific standard chemical. Assays were identified in which a significant response was detected. Assays were then ranked by AC50. Assays relevant to molecular initiating events associated with established reproductive adverse outcome pathways of fathead minnow (*Pimephales promelas*) are outlined in red.

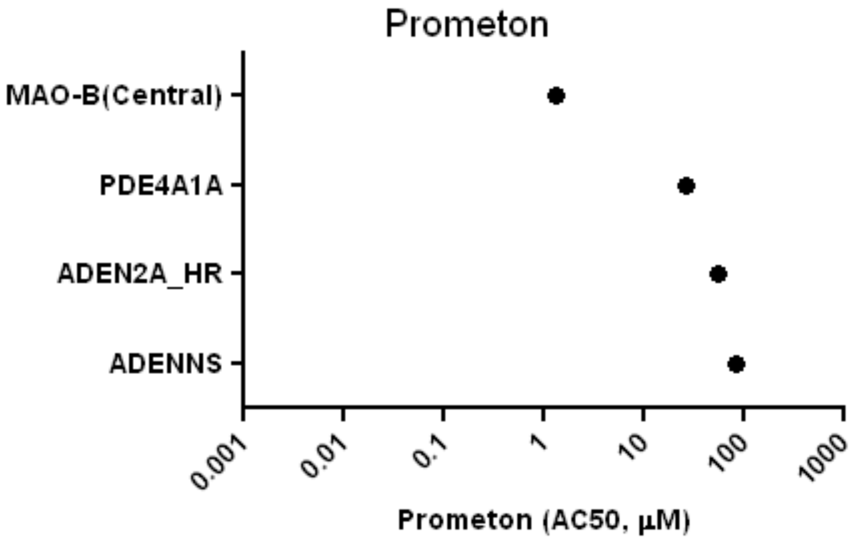

Supplemental Material, Figure S5. Molecular screening assay results for prometon tested in the ToxCast^{TM} battery of assays (Derived from Knudsen et al. 2011). An AC50 is the *in vitro* chemical concentration yielding 50% of the maximal activity produced by an assay-specific standard chemical. Assays were identified in which a significant response was detected. Assays were then ranked by AC50. Assays relevant to molecular initiating events associated with established reproductive adverse outcome pathways of fathead minnow (*Pimephales promelas*) are outlined in red.

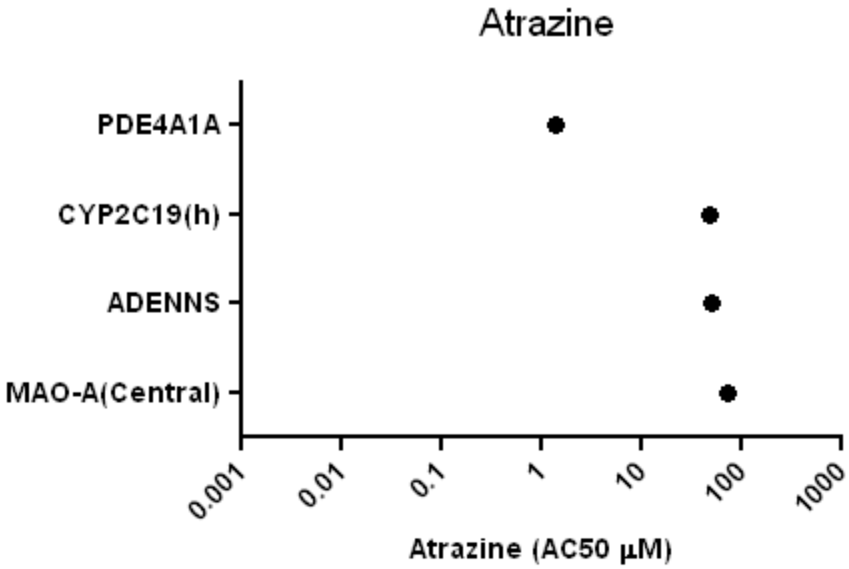

Supplemental Material, Figure S6. Molecular screening assay results for atrazine tested in the ToxCast™ battery of assays (Derived from Knudsen et al. 2011). An AC50 is the *in vitro* chemical concentration yielding 50% of the maximal activity produced by an assay-specific standard chemical. Assays were identified in which a significant response was detected. Assays were then ranked by AC50. Assays relevant to molecular initiating events associated with established reproductive adverse outcome pathways of fathead minnow (*Pimephales promelas*) are outlined in red.

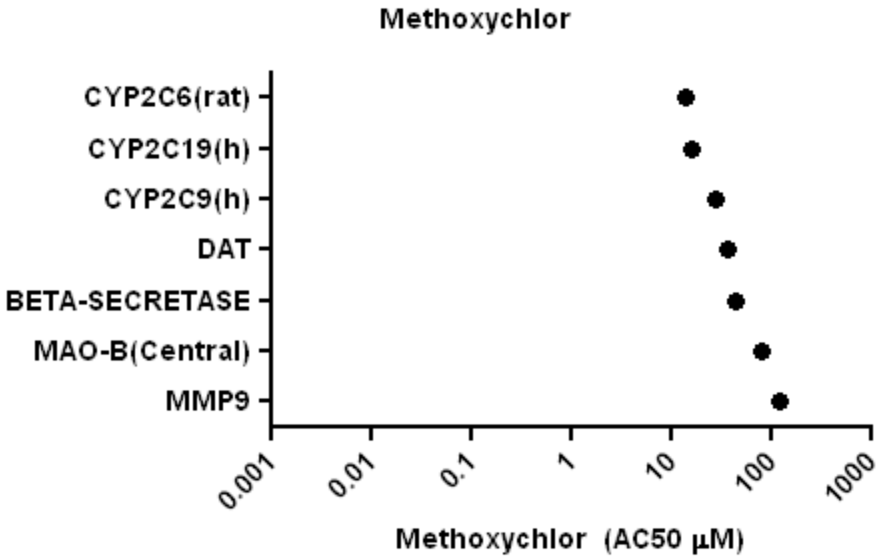

Supplemental Material, Figure S7. Molecular screening assay results for methoxychlor tested in the ToxCast^{TM} battery of assays (Derived from Knudsen et al. 2011). An AC50 is the *in vitro* chemical concentration yielding 50% of the maximal activity produced by an assay-specific standard chemical. Assays were identified in which a significant response was detected. Assays were then ranked by AC50. Assays relevant to molecular initiating events associated with established reproductive adverse outcome pathways of fathead minnow (*Pimephales promelas*) are outlined in red.

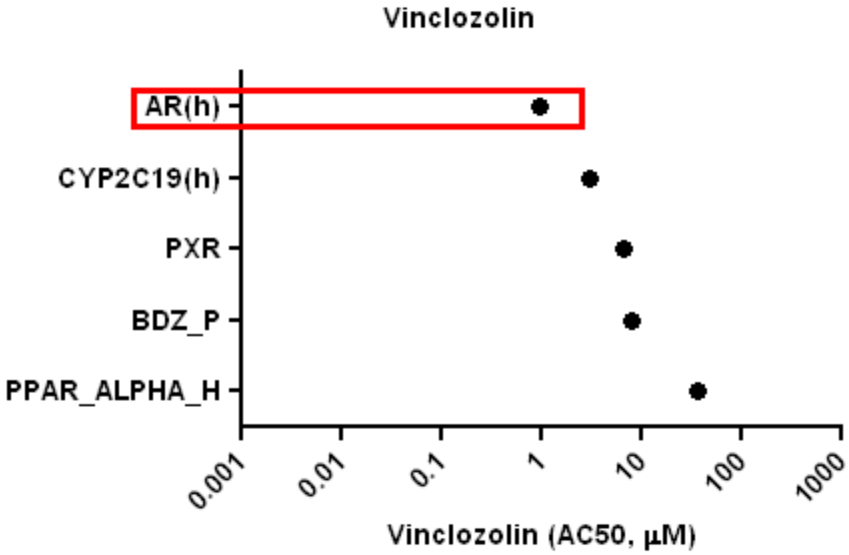

Supplemental Material, Figure S8. Molecular screening assay results for vinclozolin tested in the ToxCast^{TM} battery of assays (Derived from Knudsen et al. 2011). An AC50 is the *in vitro* chemical concentration yielding 50% of the maximal activity produced by an assay-specific standard chemical. Assays were identified in which a significant response was detected. Assays were then ranked by AC50. Assays relevant to molecular initiating events associated with established reproductive adverse outcome pathways of fathead minnow (*Pimephales promelas*) are outlined in red.

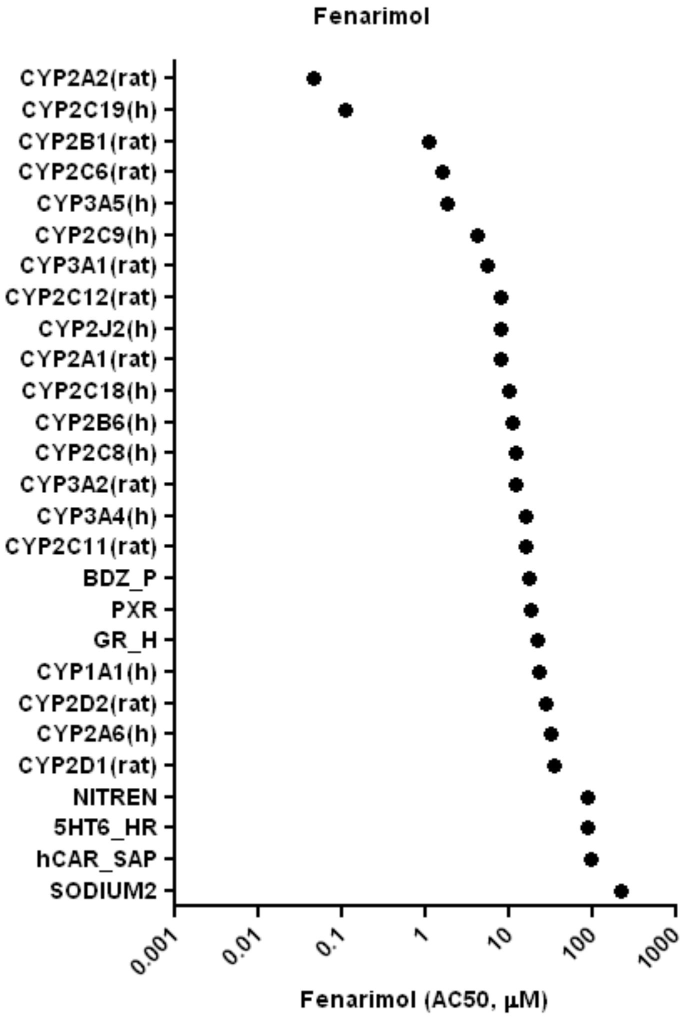

Supplemental Material, Figure S9. Molecular screening assay results for fenarimol tested in the ToxCast™ battery of assays (Derived from Knudsen et al. 2011). An AC50 is the *in vitro* chemical concentration yielding 50% of the maximal activity produced by an assay-specific standard chemical. Assays were identified in which a significant response was detected. Assays were then ranked by AC50. Assays relevant to molecular initiating events associated with established reproductive adverse outcome pathways of fathead minnow (*Pimephales promelas*) are outlined in red.

Reference

Knudsen TB, Houck KA, Sipes NS, Singh AV, Judson RS, Martin MT, et al. 2011. Activity profiles of 309 ToxCast™ chemicals evaluated across 292 biochemical targets. J Toxicol 282:1–15; doi:10.1016/j.tox.2010.12.010.
